# Elevated Lipid Infiltration Is Associated With Cerebral Aneurysm Rupture

**DOI:** 10.3389/fneur.2020.00154

**Published:** 2020-04-09

**Authors:** Chubin Ou, Yi Qian, Xin Zhang, Jiahui Liu, Wenchao Liu, Hengxian Su, Nan Zhang, Jianbo Zhang, Xuying He, Chuan-Zhi Duan

**Affiliations:** ^1^Guangdong Provincial Key Laboratory on Brain Function Repair and Regeneration, Department of Neurosurgery, National Key Clinical Specialty, Engineering Technology Research Center of Education Ministry of China, Neurosurgery Institute, Zhujiang Hospital, Southern Medical University, Guangzhou, China; ^2^Department of Biomedical Sciences, Faculty of Medicine and Health Sciences, Macquarie University, Sydney, NSW, Australia

**Keywords:** hemodynamics, intracranial aneurysm, subarachnoid hemorrhage, low-density lipoproteins, computer simulation

## Abstract

**Background:** Intracranial aneurysm wall degradation can be associated with lipid infiltration. However, the relationship between lipid infiltration and aneurysm rupture has not been explored quantitatively. To investigate the correlation between lipid infiltration and aneurysm rupture, we utilized patient-specific simulation of low-density lipoprotein (LDL) transport to analyze lipid infiltration in the cerebral aneurysm wall.

**Methods:** Sixty-two aneurysms were analyzed. Patient blood pressure, plasma LDL concentration, and three-dimensional angiographic images were obtained to simulate LDL transport in aneurysms. Morphological, hemodynamic, and lipid accumulation parameters were compared between ruptures and unruptured groups. Multivariate logistic regression was also performed to determine parameters that are independently associated with rupture.

**Results:** Size ratio, wall shear stress, low shear area, relative residence time, area-averaged LDL infiltration rate, and maximum LDL infiltration rate were significant parameters in univariate analysis (*P* < 0.05). Multivariate analysis revealed that only average LDL infiltration remained as a significant variable (*P* < 0.05). The prediction model derived showed good performance for rupture prediction (AUC, 0.885; 95% CI, 0.794–0.976).

**Conclusions:** Ruptured aneurysms showed significantly higher LDL infiltration compared to unruptured ones. Our results suggested that lipid infiltration may promote aneurysm rupture. Lipid infiltration characteristics should be considered when assessing aneurysm rupture risk.

## Background

Intracranial aneurysms were present in 3–7% of the population (1). Given the high prevalence and catastrophic consequence of rupture, early detection of aneurysms at high risk is vital. Morphological and hemodynamic parameters have been shown to be associated with aneurysm rupture (2–7), yet there is still debate on the role of wall shear stress in aneurysm rupture (8). It has also been shown that aneurysm wall degradation is associated with lipid accumulation (9–11) and the use of a lipid-lowering agent can reduce the risk of rupture (12). As aneurysm wall degradation is closely related to lipid accumulation, evaluating lipid accumulation in aneurysmal wall might provide extra information for rupture prediction. However, it is relatively difficult to measure lipid accumulation quantitatively in cerebral aneurysm *in vivo*, which limits its use in clinical settings. The simulation of low-density lipoprotein (LDL) transport in diseased arteries has demonstrated its capability in the prediction of lipid infiltration and subsequent plaque progression (13, 14). This has brought us to consider using simulation to evaluate lipid infiltration in the aneurysmal wall and its potential use in rupture prediction.

In this study, we used patient-specific simulation to evaluate the amount of LDL infiltration into the aneurysmal wall and analyzed the difference between ruptured and unruptured aneurysms. Our aim was to investigate the association between LDL infiltration and aneurysm rupture.

## Methods

### Patient Selection

The data in the current study were obtained from a single center. Approval for this study was obtained from the local institutional review board. The data were anonymous, and the requirement for informed consent was therefore waived. All patients had at least one aneurysm confirmed by three-dimensional digital subtraction angiography (DSA). Patient cases with poor image quality or incomplete record of lipid level were excluded.

### Evaluation of Morphologic Characteristics

Morphological parameters including aneurysm size, aneurysm height, aneurysm width, neck width, vessel angle, aspect ratio (AR), and size ratio (SR) were measured and calculated from three-dimensional reconstructed images according to the definition in previous research (15). Measurements were done by experienced neurosurgeons who were blinded to the status of aneurysms.

### Evaluation of Hemodynamic and LDL Infiltration Characteristics

Each aneurysm model was segmented and reconstructed using MIMICS software (Materialize). The reconstructed three-dimensional models were meshed using ANSYS ICEM CFD software (ANSYS Inc) to create a finite volume mesh composed of tetrahedral elements and prism elements at the wall boundary. Blood was assumed as incompressible Newtonian fluid governed by Navier–Stokes equations. LDL transport in blood was driven by flow convection and diffusion. The modeling of LDL transport was based on previous work, which has been validated (16, 17). LDL transport was governed by the convection–diffusion equation:

$$\begin{array}{l} \frac{\partial C}{\partial t}+U\cdot \nabla C-D\Delta C=0 \end{array}$$

where U is the blood flow velocity vector, C is the concentration of LDL, and D is the diffusion coefficient. The permeation of LDL across the endothelial membrane was through vesicular pathway and leaky junctions on endothelium. Since the distribution pattern of LDL concentration in the wall is very similar to that of LDL flux across endothelium (16), in this study, we neglected the distribution of LDL inside the wall and focused on its infiltration across the endothelium. The apparent permeability of endothelium was mediated by local wall shear stress and pressure. We used pore theory to estimate the LDL infiltration rate across the endothelium, which was calculated with local LDL concentration in blood flow, local permeability, and transmural pressure difference.

$$\begin{array}{l} \;J_{s}=(P_{v}+P_{app,lj})C\\ P_{app,lj}=P_{lj}Z_{lj}+J_{v,lj}\left(1-\sigma_{f}\right)\\ \;P_{lj}=f\left(P,WSS,a,D\right),\;J_{v,lj}=g\left(P,WSS\right) \end{array}$$

where P_app_ is the apparent permeability of the endothelium composed of P_v_, the permeability of the vesicular pathway, and P_app, lj_, the permeability of the leaky junction, which is determined by the diffusion effect P_lj_ and convection effect J_v_,_lj_. The diffusion flux P_lj_ is affected by LDL particle radius a, diffusivity D, and the area density of leaky junctions, which is dependent on local shear stress and local transmural pressure. Similarly, the convection flux J_v, lj_ is determined by the area density of leaky junctions, which is dependent on local shear stress and local transmural pressure. In summary, the LDL infiltration rate was dependent on LDL particle size, diffusivity, local shear stress, local transmural pressure, and local concentration of LDL.

The methodology was presented in detail in previous work (16–18). Physiologic pulsatile flow and patient-specific LDL concentration were prescribed at inlets, and no-slip boundary conditions were applied at vessel walls. Hemodynamic parameters including normalized time-averaged wall shear stress (WSS), normalized maximum wall shear stress (MWSS), low shear area (LSA), oscillatory shear index (OSI), and relative residence time (RRT) were calculated according to the definition presented in the literature (2). The degree of LDL infiltration was evaluated by two parameters, which are area-averaged LDL infiltration rate (LIave) and maximum LDL infiltration rate (LImax) at the aneurysm sac normalized by the physiological infiltration rate of the normal artery (19).

### Statistic Analysis

Clinical, morphological, hemodynamic, and LDL infiltration parameters were first examined by univariate analyses. Binary parameters were compared by the Fisher exact test. For continuous parameters, they were first examined by the Shapiro–Wilk test to determine if they were normally distributed. Student *t*-test (for normally distributed parameters) or Mann–Whitney *U*-test (for non-normally distributed parameters) were used to identify statistically significant parameters (*P* < 0.05) between the ruptured and unruptured group. Colinearity between variables was examined by the Pearson correlation test. Receiver operating curves were plot for each significant parameter, and their corresponding area under the curve (AUC) were calculated and compared.

Only parameters that are statistically significant and independent were considered for multivariate logistic regression. A rupture prediction model was derived using a backward stepwise method.

## Results

### Patient Demographics

Eighty-eight patients were included in the study, 33 of which were excluded due to missing data of lipid level or blood pressure or poor image quality. Among the 55 cases analyzed in this study, there were 62 aneurysms in total (45 unruptured and 17 ruptured). Among the 55 patients, 20 of them were male. The mean age was 58.

### Wall Shear Stress and LDL Infiltration Patterns

**Figure 1:**
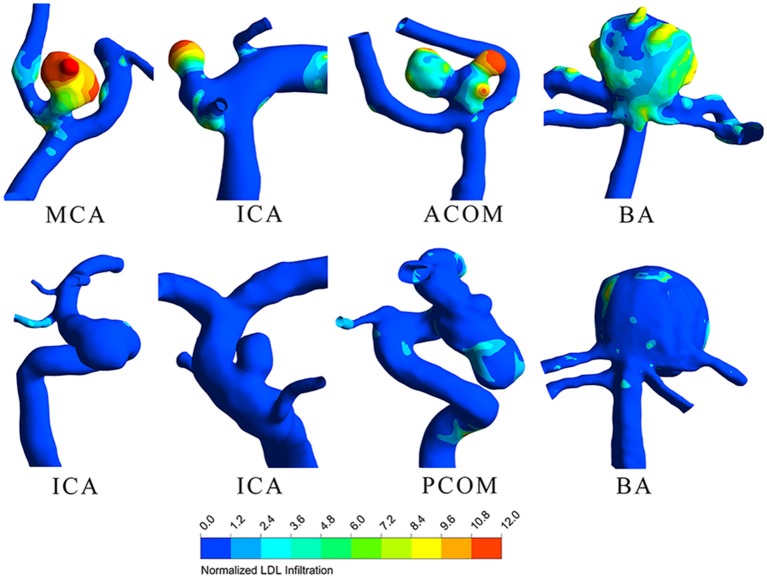
Low-density lipoprotein (LDL) infiltration distribution for four ruptured (top row) and four unruptured (bottom row) representative aneurysms.

**Figure 2:**
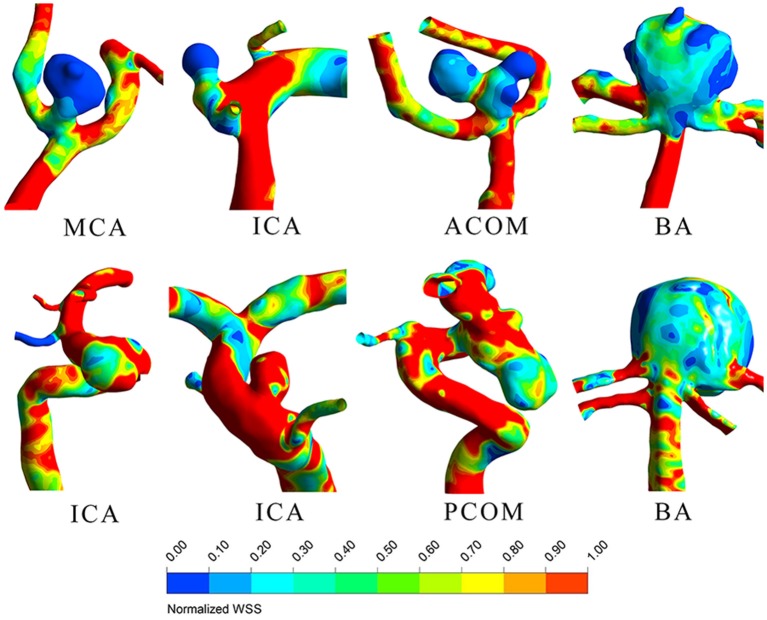
Wall shear stress distribution for four ruptured (top row) and 4 unruptured (bottom row) representative aneurysms.

LDL infiltration distribution and wall shear stress distribution for typical ruptured and unruptured aneurysms are shown in Figures 1, 2, respectively. In ruptured aneurysms, a high level of LDL infiltration can be observed in the sac especially in the area near bleb, while in unruptured aneurysms, infiltration flux in the aneurysm sac region was not much different from that in the artery region. Comparing the distribution of wall shear stress and LDL infiltration, we can see that high infiltration usually presented in the area characterized by excessively low wall shear stress, though the distribution patterns were different between the two.

### Univariate Analyses

Table 1 shows the means, standard deviations, and statistical results for each parameter.

**Table 1:** Results from univariate analysis for all parameters.

**Parameter**	**Ruptured (*n* = 17)**	**Unruptured (*n* = 45)**	***P*-value**
Age, year	57.5 ± 10.6	58.3 ± 12.1	0.716
Male sex	8 (47%)	12 (27%)	0.125
Systole, mmHg	143 ± 25	136 ± 24	0.255
Diastole, mmHg	86 ± 9	85 ± 11	0.640
Hypertension	9	12	0.051
LDL-c, mmol/L	3.02 ± 1.25	2.63 ± 0.80	0.245
Hyperlipidemia	5	6	0.139
Size, mm	5.03 ± 2.32	5.05 ± 4.30	0.372
Aneurysm height, mm	5.14 ± 1.44	5.10 ± 3.95	0.058
Aneurysm width, mm	5.24 ± 2.40	5.84 ± 5.28	0.309
Neck width, mm	4.37 ± 2.24	4.72 ± 3.34	0.813
Vessel angle, degree	114.6 ± 33.1	104.0 ± 29.5	0.273
Size ratio	2.19 ± 1.27	1.34 ± 0.91	0.006
Aspect ratio	1.49 ± 0.77	1.12 ± 0.56	0.102
WSS	0.39 ± 0.24	0.77 ± 0.40	<0.001
MWSS	1.47 ± 0.78	1.97 ± 1.10	0.116
OSI	0.033 ± 0.018	0.035 ± 0.037	0.228
RRT,s	3.44 ± 8.81	0.34 ± 0.41	<0.001
LSA	0.26 ± 0.28	0.09 ± 0.17	<0.001
LIave	4.71 ± 3.81	0.95 ± 1.39	<0.001
LImax	11.29 ± 6.48	5.91 ± 4.71	0.002

**Figure 3:**
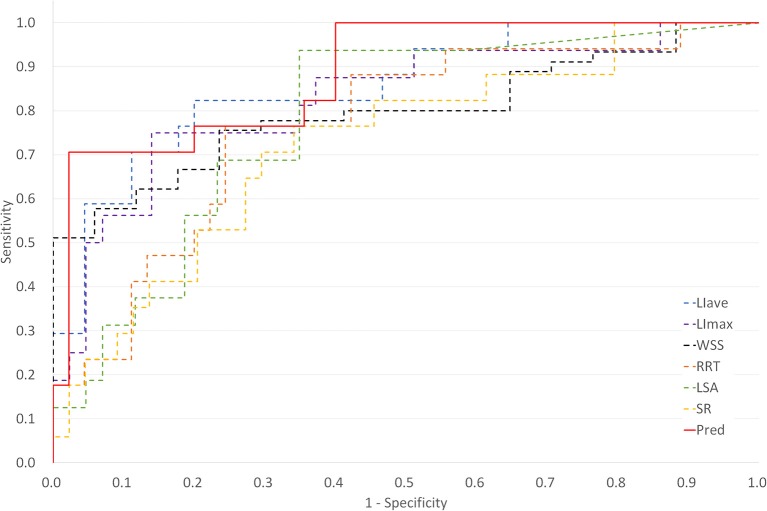
Plot of receiver operating characteristic (ROC) curves for key parameters and multivariate logistic regression derived prediction model [Pred, prediction model; LIave, area averaged LDL infiltration, area under the curve (AUC) = 0.856; LImax, maximum LDL infiltration, AUC = 0.761; WSS, wall shear stress, AUC = 0.797; RRT, relative residence time, AUC = 0.761; LSA, low shear area, AUC = 0.781; SR, size ratio, AUC = 0.725].

For LDL infiltration parameters, both LIave and LImax were significantly higher in the ruptured group than that in the unruptured groups (LIave, *P* < 0.001, LImax, *P* = 0.002). For morphological parameters, only size ratio showed a significant difference between the ruptured and unruptured cases (2.19 vs. 1.34, *P* < 0.001). For hemodynamic parameters, significant differences were observed in wall shear stress (0.39 vs. 0.77, *P* < 0.001) and low shear area (0.26 vs. 0.09, *P* < 0.001). No significant differences were observed for LDL-c plasma level, blood pressure, sex, aneurysm size, aneurysm height, neck width, inflow artery angle, aspect ratio, and OSI. Hypertension was observed to be marginally significant (*P* = 0.051) between the two groups. Receiver operator characteristic analysis was performed for parameters with significant differences, and the result is plotted in Figure 3. LIave achieved the highest area under the curve (AUC) of 0.856.

### Multivariate Regression Analyses

Colinearity between LIave, LImax, SR, WSS, LSA, and RRT were examined. Except that RRT was found to correlate with LSA (R = 0.863, *P* < 0.05), other parameters were not strongly correlated (R < 0.8). In multivariate regression analysis, LIave remained statistically significant (*P* < 0.05), as shown in Table 2. The odds ratio of LIave was 2.402 (1.237–4.665, 95% CI), which indicates that each unit increase in LDL infiltration will increase the risk by 2.4-fold. LSA and WSS were only marginally significant (*P* = 0.059, *P* = 0.071) in multivariate analysis. We derived a prediction model with LDL infiltration parameters and hemodynamic parameters. The receiver operator characteristic curves of the model is shown in Figure 3. The area under the ROC curve for the model was 0.885 (95% CI: 0.794–0.976), which indicates good discrimination between ruptured and unruptured cases.

**Table 2:** Results from multivariate analysis for key parameters.

**Parameter**	**OR**	**95% CI**	***P*-value**
LIave	2.402	1.237 to 4.665	0.010
WSS	0.040	0.001 to 1.321	0.071
LSA	0.001	0.000 to 1.286	0.059

## Discussion

Our result showed that there is significantly higher LDL infiltration at the wall of ruptured aneurysm compared to unruptured ones (4.71 vs. 0.95, *P* < 0.001). Lipid accumulation in the aneurysmal wall has been shown to be associated with aneurysm rupture (9). Accumulated lipids are oxidized, and an association between oxidized LDL and the loss of mural cells and degeneration of aneurysm wall can be found. Oxidized lipids can induce chronic inflammation in atherosclerotic lesion, which is characterized by infiltration of macrophages as in ruptured aneurysm wall. Recent research has also implicated lipid accumulation as a key factor in promoting degeneration of the aneurysm wall via formation of foam cells and subsequent loss of mural cells (10, 11). In our study, higher LDL infiltration rate was observed in the ruptured aneurysm wall, implicating lipid accumulation in ruptured aneurysms, which is consistent with the above researches. Recent advance in magnetic resonance vessel wall imaging (MR-VWI) has shown association between wall enhancement and aneurysm rupture (20). Further histopathological analyses revealed that wall enhancement was associated with wall thickening with atherosclerotic change (21, 22). Our study suggested that increased lipid infiltration was associated with rupture, which was in line with these new findings from vessel wall imaging.

In our study, we also found that LDL infiltration at the sac region can be several to 10-fold higher than normal physiological value as indicated by color in Figure 1. This suggested that given the same patient, though parent artery and aneurysm sac were exposed to the same plasma level of LDL, the actual LDL infiltration can vary at different locations. In some of our cases, even the patients' plasma LDL levels were within normal range, the infiltration rates at the aneurysm sac can still be much higher, which infers lipid accumulation. Our finding agrees with previous research that lipid accumulation was observed in the aneurysm wall despite normal plasma lipid level (9).

We further observed that lipid infiltration pattern was quite heterogenous at the sac region. Higher infiltration tended to appear at the tip or at blebs, as shown in Figure 1. As higher infiltration can lead to more extensive lipid accumulation, oxidation, and subsequent degradation of the wall at the local spot, this may explain why the rupture sites of aneurysm are usually located at the tip or bleb. The heterogenous pattern of LDL infiltration was, in part, owing to spatially varying permeability of the vessel wall, which was dependent on the local wall shear stress. We compared Figures 1, 2 and found out that high infiltration regions were associated with excessively low wall shear stress regions, which is consistent with literature studies that low shear stress was associated with atherosclerotic change in the aneurysm wall (23–26). Though aneurysm initiation is generally considered to be linked with high shear stress (27, 28), the role of wall shear stress in aneurysm rupture remains controversial. In some studies, the rupture sites of aneurysms were found to be associated with the low shear stress region (29, 30). Meng et al. proposed that aneurysm rupture may follow two different pathways: one is mural cell-mediated pathway and the other is inflammatory cell-mediated pathway (31). As lipid infiltration has been recognized as the trigger for inflammation in atherosclerosis, it is possible that lipid accumulation may also promote inflammation process leading to aneurysm wall degradation and rupture.

Besides local shear stress, transmural pressure, and LDL plasma level also affect the amount of LDL infiltration. They acted as global and systemic factors on LDL accumulation, while shear stress acted as a local mediator. Lipid accumulation was the combined effect from both local factor and global factors. For example, patients having a high lipid level or high blood pressure does not necessarily indicate a high lipid accumulation in the aneurysm wall and vice versa. LDL, though widely recognized as risk factors for cardiovascular and cerebrovascular disease, was not found to be associated with cerebral aneurysm rupture in a recent clinical study (12), which seems to conflict with findings from a histopathology study (9, 10). However, it is important to note that despite LDL not recognized as a risk factor, the use of a lipid-lowering agent was found to be able to reduce the risk of rupture (12). This seemly contradiction can be explained by the intermingled effect of global factors (LDL plasma level) and local factors (WSS). As demonstrated in our result, the LDL plasma level alone cannot represent the local infiltration of LDL at the aneurysm sites, which explains why LDL plasma level did not differ significantly between ruptured and unruptured aneurysms in clinical study. Wall shear stress pattern can vary greatly between aneurysms from different patients. However, if focusing on a single patient, local factors such as shear stress will remain largely stable, the use of lipid-lowering agent can reduce plasma lipid level and alleviate lipid accumulation (by changing the global factors), therefore reducing the risk of rupture, as evident in clinical study (12). Our result can help to reconcile the different conclusions obtained from clinical study and histopathology studies.

Since the permeability of the endothelium was linked to shear stress in the model, though there was no colinearity between LIave and WSS, there is an association between these two parameters (r = 0.59, *P* < 0.001). However, since LIave was calculated with additional information from patient-specific blood pressure and plasma LDL level, it appeared to perform better than WSS alone in discriminating aneurysm rupture, as illustrated by the higher AUC achieved by LIave than in the WSS and other hemodynamic parameters.

### Limitations

All the cases were from a single center, and the number of cases is small. More cases from multiple centers should be analyzed to verify the finding. The current study was retrospective in which morphology of post-rupture aneurysms may be different from their pre-rupture shapes (32). For unruptured aneurysms, they remained unruptured during follow-up. The number of ruptured aneurysms was smaller than that of unruptured ones, which may introduce bias to our study.

As for the modeling of hemodynamics and LDL transport, there were several assumptions made. We assumed a generic inflow waveform scaled by vessel diameters. In the model, we only considered LDL infiltration through endothelium, which is the first step of lipid accumulation. Owing to the difficulty in accurate measurement of aneurysm wall thickness, a uniform thickness was assumed (33). Nevertheless, to our best knowledge, this is the first study utilizing patient-specific modeling of LDL transport to examine the relationship between lipid transport and cerebral aneurysm rupture. An *ex vivo* histology study on resected aneurysm tissue should be conducted to validate the simulation model. Further, we can compare the simulation results with high-resolution MR-VWI images to investigate the correlation between the LDL infiltration zone and image enhancement region.

## Conclusions

We have investigated the association between lipid accumulation and cerebral aneurysm rupture using patient-specific modeling of LDL transport. Ruptured aneurysms had significantly higher LDL infiltration than unruptured ones, which suggested that lipid accumulation may promote aneurysm rupture. Lipid accumulation characteristics should be considered when assessing cerebral aneurysm rupture risk.

## Data Availability Statement

The datasets used during the current study are available from the corresponding author on reasonable request.

## Ethics Statement

The studies involving human participants were reviewed and approved by Zhujiang Hospital. Written informed consent for participation was not required for this study in accordance with the national legislation and the institutional requirements.

## Author Contributions

All authors drafted the manuscript. CO and YQ performed the simulation and analysis. XZ, JL, WL, HS, NZ, and JZ collected the data and performed data preprocessing. XH and C-ZD supervised the study.

### Conflict of Interest

The authors declare that the research was conducted in the absence of any commercial or financial relationships that could be construed as a potential conflict of interest.

## Abbreviations

LDL: low-density lipoprotein
WSS: normalized wall shear stress
MWSS: maximum normalized wall shear stress
OSI: oscillatory shear index
LSA: low shear area
RRT: relative residence time
CFD: computational fluid dynamics
AR: aspect ratio
SR: size ratio
LIave: area-averaged normalized lipid infiltration rate
LImax: maximum normalized lipid infiltration rate
ROC: receiver operating characteristic curve
AUC: area under the curve.

## References

[1] Li MH Chen SW Li YD Chen YC Cheng YS Hu DJ. Prevalence of unruptured cerebral aneurysms in Chinese adults aged 35 to 75 years: a cross-sectional study. Ann Intern. (2013) 159:514–21. 10.7326/0003-4819-159-8-201310150-00004 24126645

[2] Xiang J Natarajan SK Tremmel M Ma D Mocco J Hopkins LN. Hemodynamic–morphologic discriminants for intracranial aneurysm rupture. Stroke. (2011) 42:144–52. 10.1161/STROKEAHA.110.592923 21106956 PMC3021316

[3] Varble N Tutino VM Yu J Sonig A Siddiqui AH Davies JM. Shared and distinct rupture discriminants of small and large intracranial aneurysms. Stroke. (2018) 49:856–64. 10.1161/STROKEAHA.117.019929 29535267 PMC5871584

[4] Cebral JR Mut F Weir J Putman C. Quantitative characterization of the hemodynamic environment in ruptured and unruptured brain aneurysms. AJNR Am J Neuroradiol. (2011) 32:145–51. 10.3174/ajnr.A2419 21127144 PMC3086563

[5] Takao H Murayama Y Otsuka S Qian Y Mohamed A Masuda S. Hemodynamic differences between unruptured and ruptured intracranial aneurysms during observation. Stroke. (2012) 43:1436–9. 10.1161/STROKEAHA.111.640995 22363053

[6] Zhang X Karuna T Yao ZQ Duan CZ Wang XM Jiang ST High wall shear stress beyond a certain range in the parent artery could predict the risk of anterior communicating artery aneurysm rupture at follow-up. J Neurosurg. (2018) 1:1–8. 10.3171/2018.4.JNS173179 30265195

[7] Miura Y Ishida F Umeda Y Tanemura H Suzuki H Matsushima S. Low wall shear stress is independently associated with the rupture status of middle cerebral artery aneurysms. Stroke. (2013) 44:519–21. 10.1161/STROKEAHA.112.675306 23223503

[8] Zhou G Zhu Y Yin Y Su M Li M. Association of wall shear stress with intracranial aneurysm rupture: systematic review and meta-analysis. Sci Rep. (2017) 7:5331. 10.1038/s41598-017-05886-w 28706287 PMC5509692

[9] Frösen J Tulamo R Heikura T Sammalkorpi S Niemela M Hernesniemi J. Lipid accumulation, lipid oxidation, and low plasma levels of acquired antibodies against oxidized lipids associate with degeneration and rupture of the intracranial aneurysm wall. Acta Neuropathol Commun. (2013) 1:71. 10.1186/2051-5960-1-71 24252658 PMC3893371

[10] Ollikainen E Tulamo R Lehti S Lee-Rueckert M Hernesniemi J Niemela M. Smooth muscle cell foam cell formation, apolipoproteins, and ABCA1 in intracranial aneurysms: implications for lipid accumulation as a promoter of aneurysm wall rupture. J Neuropath Exp Neur. (2016) 75:689–99. 10.1093/jnen/nlw041 27283327 PMC4913436

[11] Ollikainen E Tulamo R Lehti S Hernesniemi J Niemela M Kovanen PT. Myeloperoxidase associates with degenerative remodeling and rupture of the saccular intracranial aneurysm wall. J Neuropath Exp Neur. (2018) 77:461–8. 10.1093/jnen/nly028 29718300

[12] Can A Castro VM Dligach D Finan S Yu S Gainer V. Lipid-lowering agents and high HDL (high-density lipoprotein) are inversely associated with intracranial aneurysm rupture. Stroke. (2018) 49:1148–54. 10.1161/STROKEAHA.117.019972 29622625 PMC5915939

[13] Sakellarios A Bourantas CV Papadopoulou SL Tsirka Z de Vries T Kitslaar PH. Prediction of atherosclerotic disease progression using LDL transport modelling: a serial computed tomographic coronary angiographic study. Eur Heart J Cardiovasc Imaging. (2016) 18:11–8. 10.1093/ehjci/jew035 26985077

[14] Sakellarios AI Räber L Bourantas CV Exarchos TP Athanasiou LS Pelosi G. Prediction of atherosclerotic plaque development in an *in vivo* coronary arterial segment based on a multilevel modeling approach. IEEE Trans Bio Med Eng. (2016) 64:1721–30. 10.1109/TBME.2016.2619489 28113248

[15] Dhar S Tremmel M Mocco J Yamamoto J Siddiqui AH Hopkins LN. Morphology parameters for intracranial aneurysm rupture risk assessment. Neurosurgery. (2008) 63:185–97. 10.1227/01.NEU.0000316847.64140.81 18797347 PMC2570753

[16] Olgac U Poulikakos D Saur SC Alkadhi H Kurtcuoglu V. Patient-specific three-dimensional simulation of LDL accumulation in a human left coronary artery in its healthy and atherosclerotic states. Am J Physiol Heart Circ. (2009) 296:H1969–82. 10.1152/ajpheart.01182.2008 19329764

[17] Olgac U Knight J Poulikakos D Saur SC Alkadhi H Desbiolles LM. Computed high concentrations of low-density lipoprotein correlate with plaque locations in human coronary arteries. J Biomech. (2011) 44:2466–71. 10.1016/j.jbiomech.2011.06.022 21723556

[18] Jesionek K Slapik A Kostur M. Low-density lipoprotein transport through an arterial wall under hypertension–a model with time and pressure dependent fraction of leaky junction consistent with experiments. J Theor Biol. (2016) 411:81–91. 10.1016/j.jtbi.2016.09.020 27686595

[19] Deng X Marois Y How T Merhi Y King M Guidoin R. Luminal surface concentration of lipoprotein (LDL) and its effect on the wall uptake of cholesterol by canine carotid arteries. J Vasc Surg. (1995) 21:135–45. 10.1016/S0741-5214(95)70252-0 7823352

[20] Edjlali M Gentric JC Régent-Rodriguez C Trystram D Hassen WB Lion S. Does aneurysmal wall enhancement on vessel wall MRI help to distinguish stable from unstable intracranial aneurysms? Stroke. (2014) 45:3704–6. 10.1161/STROKEAHA.114.006626 25325912

[21] Shimonaga K Matsushige T Ishii D Sakamoto S Hosogai M Kawasumi T. Clinicopathological insights from vessel wall imaging of unruptured intracranial aneurysms. Stroke. (2018) 49:2516–9. 10.1161/STROKEAHA.118.021819 30355091

[22] Hudson JS Zanaty M Nakagawa D Kung DK Jabbour P Samaniego EA Magnetic resonance vessel wall imaging in human intracranial aneurysms: histological analysis. Stroke. (2019) 50:e1 10.1161/STROKEAHA.118.023701 30580739

[23] Sugiyama S-i Niizuma K Nakayama T Shimizu H Endo H Inoue T. Relative residence time prolongation in intracranial aneurysms: a possible association with atherosclerosis. Neurosurgery. (2013) 73:767–76. 10.1227/NEU.0000000000000096 23863763

[24] Jiang P Liu Q Wu J Chen X Li M Yang F. Hemodynamic findings associated with intraoperative appearances of intracranial aneurysms. Neurosurg Rev. (2018) 43:1–7. 10.1007/s10143-018-1027-0 30242546

[25] Cebral JR Detmer F Chung BJ Choque-Velasquez J Rezai B Lehto H. Local hemodynamic conditions associated with focal changes in the intracranial aneurysm wall. AJNR Am J Neuroradiol. (2019) 40:510–6. 10.3174/ajnr.A5970 30733253 PMC6420361

[26] Tateshima S Tanishita K Omura H Sayre J Villablanca JP Martin N. Intra-aneurysmal hemodynamics in a large middle cerebral artery aneurysm with wall atherosclerosis. Surg Neurol. (2008) 70:454–62. 10.1016/j.surneu.2008.03.035 18514767

[27] Kulcsár Z Ugron A Marosfoi M Berentei Z Paál G Szikora I. Hemodynamics of cerebral aneurysm initiation: the role of wall shear stress and spatial wall shear stress gradient. AJNR Am J Neuroradiol. (2011) 32:587–94. 10.3174/ajnr.A2339 21310860 PMC8013095

[28] Zhang X Yao Z-Q Karuna T He XY Wang XM Li XF. The role of wall shear stress in the parent artery as an independent variable in the formation status of anterior communicating artery aneurysms. Eur Radiol. (2019) 29:689–98. 10.1007/s00330-018-5624-7 30019140

[29] Kadasi LM Dent WC Malek AM. Colocalization of thin-walled dome regions with low hemodynamic wall shear stress in unruptured cerebral aneurysms. J Neurosurg. (2013) 119:172–9. 10.3171/2013.2.JNS12968 23540271

[30] Suzuki T Stapleton CJ Koch MJ Tanaka K Fujimura S Suzuki T Decreased wall shear stress at high-pressure areas predicts the rupture point in ruptured intracranial aneurysms. J Neurosurg. (2019) 1:1–7. 10.3171/2018.12.JNS182897 30875692

[31] Meng H Tutino V Xiang J Siddiqui A. High WSS or low WSS? Complex interactions of hemodynamics with intracranial aneurysm initiation, growth, and rupture: toward a unifying hypothesis. AJNR Am J Neuroradiol. (2014) 35:1254–62. 10.3174/ajnr.A3558 23598838 PMC7966576

[32] Skodvin TØ Johnsen L-H Gjertsen Ø Isaksen JG Sorteberg A. Cerebral aneurysm morphology before and after rupture: nationwide case series of 29 aneurysms. Stroke. (2017) 48:880–6. 10.1161/STROKEAHA.116.015288 28265012

[33] Torii R Oshima M Kobayashi T Takagi K Tezduyar TE Numerical investigation of the effect of hypertensive blood pressure on cerebral aneurysm—dependence of the effect on the aneurysm shape. Int J Numer Methods Fluids. (2007) 54:995–1009. 10.1002/fld.1497

